# Microbiome-mediated modulation of immune memory to *P. yoelii* affects the resistance to secondary cerebral malaria challenge

**DOI:** 10.1093/immhor/vlaf009

**Published:** 2025-03-28

**Authors:** Elizabeth M Fusco, Layne Bower, Rafael Polidoro, Allen M Minns, Scott E Lindner, Nathan W Schmidt

**Affiliations:** Department of Microbiology and Immunology, Indiana University School of Medicine, Indianapolis, IN, United States; Herman B. Wells Center for Pediatric Research, Indiana University School of Medicine, Indianapolis, IN, United States; Ryan White Center for Pediatric Infectious Diseases and Global Health, Indiana University School of Medicine, Indianapolis, IN, United States; Herman B. Wells Center for Pediatric Research, Indiana University School of Medicine, Indianapolis, IN, United States; Ryan White Center for Pediatric Infectious Diseases and Global Health, Indiana University School of Medicine, Indianapolis, IN, United States; The Huck Institutes of Life Sciences, Pennsylvania State University, University Park, PA, United States; Department of Biochemistry and Molecular Biology, Pennsylvania State University, University Park, PA, United States; The Huck Center for Malaria Research, University Park, PA, United States; The Huck Institutes of Life Sciences, Pennsylvania State University, University Park, PA, United States; Department of Biochemistry and Molecular Biology, Pennsylvania State University, University Park, PA, United States; The Huck Center for Malaria Research, University Park, PA, United States; Department of Microbiology and Immunology, Indiana University School of Medicine, Indianapolis, IN, United States; Herman B. Wells Center for Pediatric Research, Indiana University School of Medicine, Indianapolis, IN, United States; Ryan White Center for Pediatric Infectious Diseases and Global Health, Indiana University School of Medicine, Indianapolis, IN, United States

**Keywords:** malaria, plasmodium, gut microbiome, germinal centers, antibodies

## Abstract

Malaria is caused by protozoan parasites in the genus *Plasmodium*. Over time individuals slowly develop clinical immunity to malaria, but this process occurs at variable rates, and the mechanism of protection is not fully understood. We have recently demonstrated that in genetically identical C57BL/6N mice, gut microbiota composition dramatically impacts the quality of the humoral immune response to *Plasmodium yoelii* and subsequent protection against a lethal secondary challenge with *Plasmodium berghei* ANKA in C57BL/6N mice. Here, we utilize this genetically identical, gut microbiome–dependent model to investigate how the gut microbiota modulate immunological memory, hypothesizing that the gut microbiome impacts the formation and functionality of immune memory. In support of this hypothesis, *P. yoelii* hyperparasitemia–resistant C57BL/6N mice exhibit increased protection against *P. berghei* ANKA–induced experimental cerebral malaria (ECM) compared to *P. yoelii* hyperparasitemia–susceptible C57BL/6N mice. Despite differences in protection against ECM, *P. yoelii*–resistant and –susceptible mice accumulate similar numbers of memory B cells (MBCs) and memory T cells. Following challenge with *P. berghei* ANKA, *P. yoelii*–resistant mice generated more rapid germinal center reactions; however, *P. yoelii*–resistant and –susceptible mice had similar titers of *P. yoelii*– and *P. berghei*–specific antibodies. In contrast, *P. yoelii*–resistant mice had an increased number of regulatory T cells in response to secondary challenge with *P. berghei* ANKA, which may dampen the immune-mediated breakdown of the blood–brain barrier and susceptibility to *P. berghei*–induced ECM. These findings demonstrate the ability of the gut microbiome to shape immune memory and the potential to enhance resistance to severe malaria outcomes.

## Introduction

More than 40% of the world lives in malaria-endemic regions, and the parasite that causes malaria, *Plasmodium* spp., is quickly developing resistance against current therapies and treatments. Vaccines against *Plasmodium* are estimated to have between 60% to 75% efficacy 12 months after administration; however, this efficacy continues to drop several years after administration.^1–5^ Decreasing efficiency over time is potentially because these vaccines fail to generate functional memory B cells and long-lived plasma cells that are specific to *Plasmodium falciparum* in humans living in malaria-endemic regions. The blood-stage of *Plasmodium* infection causes the clinical manifestation of malaria, including fever, chills, and anemia.^6^ In severe cases, *P. falciparum* can impact the brain and cause neurological symptoms, coma, and even death in a complication known as cerebral malaria.^7^^,^^8^ There are many challenges associated with studying the immune response to malaria in humans. Consequently, mouse models of malaria are used to study the immune response to *Plasmodium*. *Plasmodium yoelii* 17XNL (*P. yoelii*) and *Plasmodium berghei* ANKA induce symptoms in C57BL/6 mice that mimic what is seen in *P. falciparum* infection in humans.^9^ Specifically, *P. yoelii* is a nonlethal model used to induce hyperparasitemia, and *P. berghei* ANKA is a lethal model used to study experimental cerebral malaria (ECM) and hyperparasitemia.^9^ Unlike humans who remain susceptible to repeat *Plasmodium* infections, mice generate sterilizing immunity to *P. yoelii* after just one exposure, so a cross-species *Plasmodium* challenge is used to study the immune response to reinfection. We accomplish this by infecting mice with *P. yoelii* and challenging them with *P. berghei* ANKA. By utilizing these models, we can use *P. yoelii* to study the formation of immune memory, and *P. berghei* ANKA to study the functionality of immune memory to control the *Plasmodium* parasite and to establish clinical immunity.

The humoral immune system plays a large role in the clearance of *Plasmodium* infection and protection from future infection.^9–12^ *Plasmodium* infections induce germinal center (GC) reactions, which are the site of antibody somatic hypermutation and affinity maturation, production of GC-derived memory B cells (MBCs), and antibody-producing plasma cells (PCs).^13^^,^^14^ Impairing GC formation results in higher *P. yoelii* parasite burden and prolonged clearance.^15^^,^^16^ After generation, PCs migrate to the bone marrow, where they reside and continuously secrete antigen-specific antibodies to protect against reinfection.^17^ Upon reinfection, MBCs help activate the secondary GC response and have the potential to differentiate further into PCs.^17–20^ Memory T cells also play an important role in preventing malaria symptoms and activating the secondary GC response, but little is also known about their role in protecting against *Plasmodium* reinfection.^21^^,^^22^ Better characterization of these cell types and their function will help bridge the gap in the development of a more effective, long-lasting malaria vaccine.

An emerging modulator of the immune response is the gut microbiome. The gut microbiome is the collection of microorganisms and their genetic content that reside within the gastrointestinal tract. Research has demonstrated that gut microbiota influence the function of the immune system in several viral diseases, but aside from this very little is known about the link between the gut microbiome and the immune response to non-intestinal infections.^23–28^ Importantly, we have demonstrated through multiple approaches, including fecal microbiota transplants into germ-free mice, that gut bacteria can cause susceptibility to *P. yoelii* hyperparasitemia.^29–31^ Consistent with these reports, and others, there are distinct differences in the gut microbiome of genetically similar mice from different vendors, and even within different barrier rooms of the same vendor.^29^^,^^30^^,^^32–34^ C57BL/6 mice from specific barrier rooms at Taconic Biosciences (Tac) are protected from *P. yoelii* hyperparasitemia, while C57BL/6 mice from Charles River Laboratories (CR) are susceptible to *P. yoelii* hyperparasitemia.^29^^,^^33^ We have also demonstrated that the composition of the gut microbiome is associated with the susceptibility of children to severe malaria anemia.^30^^,^^31^ Additionally, we discovered that gut microbiota impact the generation of immune effector cells during infection with *P. yoelii* and the ability to survive secondary challenge with *P. berghei* ANKA.^33^ We hypothesize that the composition of the gut microbiome influences the formation and functionality of immune memory in the context of a *Plasmodium* infection. We aim to further characterize how the gut microbiome impacts the functionality of immunological memory to *Plasmodium* and how this impacts the secondary effector response to *Plasmodium* challenge.

## Materials and methods

### Mice

Conventional female C57BL/6N mice (6 to 8 weeks old) were purchased from Charles River Laboratories (Barrier Room R01; Hollister, California) and Taconic Biosciences (Barrier Room IBU504; Cambridge City, Indiana). Mice were given a minimum of 6 d to acclimate prior to the beginning of experiments. All mice were fed Teklad 7913 Mouse/Rat Irradiated Diet and non-acidified water. All animal experiments were carried out at the Indiana University School of Medicine adhering to the local and national regulations of laboratory animal welfare, and all procedures were reviewed and approved by the university’s Institutional Animal Care and Use Committees (IACUC protocol numbers 19024 and 22010).

### *Plasmodium* infections

Mice were infected with *P. yoelii* 17XNL (BEI Resources/MR4/ATCC) and/or *P. berghei* ANKA (BEI Resources/MR4/ATCC) through intravenous injection of 1.5 × 10^5^ parasitized red blood cells prepared from fresh donor blood. In secondary challenge experiments *P. yoelii*–immune mice were infected with *P. berghei* ANKA on d 60 post–*P. yoelii* infection, and parasitemia was monitored starting at d 4 or 5 post–*P. berghei* infection (d 64 post–initial infection with *P. yoelii*). On d 6 to 8 postinfection (p.i.) with *P. berghei* ANKA, mice were monitored for the development of experimental cerebral malaria by evaluating gait, balance, motor performance, body position, limb strength, touch escape, pinna reflex, toe touch, and grooming, given a score between 0 and 18. A score of 10 or less resulted in humane euthanasia. With IACUC approval, several mice were kept alive for additional observation and experimentation after receiving a score of less than 10, but these mice were all humanely euthanized at later time points. After d 9 p.i. with *P. berghei* ANKA, parasitemia was monitored for the development of hyperparasitemia (>60% parasitemia) until either parasite clearance or until the endpoint was reached.

### Evaluation of parasitemia

Blood samples were taken through tail snips at regular intervals ranging from d 4 to 30 p.i. Percent parasitemia was determined as the number of infected red blood cells per total red blood cells, and this was assessed through either blood smear slides or flow cytometry. For blood smear evaluation, blood smears were fixed and stained using HARLECO Hemacolor solution (EMD Millipore, Burlington, Massachusetts), and parasitemia was determined by light microscopy counting >300 red blood cells per slide at 1000× magnification. For flow cytometry evaluation, a drop of blood was added to FACS buffer and stained with CD45.2-APC (clone 104; BioLegend, San Diego, California), Ter119-APC/Cy7 (clone TER-119; BioLegend, San Diego, California), dihydroethidium (Sigma-Aldrich, St. Louis, Missouri), and Hoechst 33342 (Sigma-Aldrich; St. Louis, Missouri). Parasitized red blood cells were defined as CD45.2^−^ Terr119^+^ Dihydroethidium^+^ Hoechst^+^ cells.

### Immunophenotyping

Spleens and/or bones were harvested from mice at the indicated day p.i. Spleens were smashed through screens in RP-10 media to generate a single-cell suspension. RP-10 was generated by supplementing Hyclone RPMI Medium 1640 (GIBCO, Thermo Fisher Scientific, Waltham, Massachusetts) with 10% v/v FBS (Atlanta Biologicals, Inc., Lawrenceville, Georgia), 1.19 mg/ml HEPES (Thermo Fisher Scientific, Waltham, Massachusetts), 0.2 mg/ml L-glutamine (Research Products International Corp., Mt. Prospect, Illinois), 0.05 units/ml and 0.05 mg/ml (respectively) penicillin/streptomycin (Invitrogen, Grand Island, New York), 0.05 mg/ml gentamicin sulfate (Invitrogen, Grand Island, New York), and 0.05 mM 2-mercaptoethanol (Thermo Fisher Scientific Inc., Waltham, Massachusetts). Femurs and tibias were harvested from mice on the indicated day p.i. Bone marrow was collected from the bone by removing the caps and flushing the bone shaft with RP-10 media. A single-cell suspension was achieved by smashing the marrow through a 70-µM cell strainer (CellTreat, Pepperell, Massachusetts). Single-cell suspensions were treated with ammonium chloride potassium to lyse red blood cells. Cells were stained with Zombie Aqua Fixable Viability Kit (BioLegend, San Diego, California) for 20 min at 4 °C. Cells were then incubated with Fc block (anti-CD16/32; clone 2.4G2) for 10 min at 4 °C, and extracellularly stained for 30 min at 4 °C with antibodies resuspended in FACS buffer (1× PBS, 0.02% w/v sodium azide, and 1% v/v FCS). This was followed by fixation with fixation buffer (BioLegend, San Diego, California).

For intracellular staining, the eBioscience Foxp3/Transcription Factor Staining Buffer Set (Thermo Fisher, Waltham, Massachusetts) was used according to manufacturer’s instructions. All samples were collected using an Attune NxT (Thermo Fisher, Waltham, Massachusetts) and analyzed by FlowJo software (Tree Star, Ashland, OR).

### MSP1_19_ production

Expression plasmid pSL1331 was created by ligating the synthesized coding sequences for PyMSP1_19_ (AA1619-1754), which was purchased from IDT as gBlock, into a modified pET28 vector, pSL1327, which incorporates an N-terminal GST and a SpyTag and 6xHis on the C-terminus. The expression plasmid was transformed into BL21 CodonPlus (DE3) and cultured into LB media at 23 °C. When cultures reached OD600 of ∼0.5, protein expression was induced by the addition of IPTG to the media at a final concentration of 0.5 mM, and the cultures were harvested 16 h later. Cell pellets were suspended in 50 mL of low-imidazole buffer (25 mM Tris-Cl pH 7.5 at room temperature (RT), 500 mM NaCl, 10 mM imidazole, and 10% glycerol). To lyse the cells, the suspension was sonicated 3 times, where each time was 30 s at 70% amplitude and 50% duty cycle. The crude extract was spun 15,500 × *g* for 20 min at 4 °C. The soluble lysate was then incubated with 2 mL of equilibrated Ni-NTA resin (Thermo Scientific, cat. # 88223) for 1 h at 4 °C. The resin was applied to a gravity column and washed with 50 mL of low-imidazole buffer followed by 50 mL of mid-imidazole (25 mM Tris-Cl pH 7.5 at RT, 500 mM NaCl, 50 mM imidazole, 250 mM dextrose, and 10% glycerol). PyMSP1_19_ was then eluted using high-imidazole buffer (25 mM Tris-Cl pH 7.5 at RT, 500 mM NaCl, 300 mM imidazole, and 10% glycerol). The elution pool was dialyzed overnight into 20 mM Tris-Cl pH 8.0 at RT, 100 mM NaCl, 1 mM DTT, and 10% glycerol. The sample was then purified further using anion exchange chromatography by applying the sample to a 20-mL Q-Sepharose column that was equilibrated in buffer A (20 mM Tris-Cl pH 8.0 at RT, 50 mM NaCl, 1 mM DTT, and 10% glycerol). Column was washed with several column volumes of buffer A. Sample was eluted using a linear gradient of 0% to 100% buffer B (20 mM Tris-Cl pH 8.0 at RT, 1000 mM NaCl, 1 mM DTT, and 10% glycerol) over 10 column volumes. The elution fractions were pulled and exhaustively dialyzed into 50 mM Tris-Cl pH 8.0 at RT, 100 mM NaCl, 1 mM DTT, and 10% glycerol. The dialyzed material was then concentrated to ∼2.0 mg/mL using Amicon Ultra Centrifugal Filter.

### ELISA

For detection of *Plasmodium*-specific antibodies, blood was collected through retro-orbital bleed on the indicated days p.i., allowed to clot for at least 30 min, and centrifuged for 10 min at 1,000 RCF to collect serum. Serum was stored at –80 °C until use.

To evaluate MSP1_19_-specific antibodies, MaxiSorp Immuno plates (Thermo Fisher Scientific, Waltham, Massachusetts) were coated with 10 µg/ml recombinant MSP1_19_ overnight at 4 °C. Plates were blocked for 2 h at RT with 2.5% w/v BSA + 5% v/v FCS in PBS. Dilutions of serum were added to wells and incubated overnight. Horseradish peroxidase–conjugated goat anti-mouse IgM (Jackson ImmunoResearch, West Grove, Pennsylvania), goat anti-mouse IgG (Jackson ImmunoResearch, West Grove, Pennsylvania), goat anti-mouse IgG1 (Jackson ImmunoResearch, West Grove, Pennsylvania), or goat anti-mouse IgG2b (Jackson ImmunoResearch, West Grove, Pennsylvania) were added and incubated for 1 h at RT. Plates were developed with a TMB substrate set (BioLegend, San Diego, California). Two moles H_2_SO_4_ was used to halt the reaction, and plates were read using a microplate reader with the absorbance read at an absorbance 450 nm.

To evaluate bulk whole parasite lysate–specific IgM, IgG1, IgG2b, and IgG, *Plasmodium* whole parasites were isolated from whole blood of *Plasmodium yoelii* 17XNL– or *Plasmodium berghei* ANKA–infected mice using a 35%/65% Percoll gradient. Infected red blood cells were then lysed, and protein concentration was calculated using a Bradford Protein Assay (Thermo Fisher Scientific, Waltham, Massachusetts). The ELISA assay was then performed as noted above.

To evaluate antibody-binding affinity, blood was collected through retro-orbital bleed on the indicated days p.i., allowed to clot for at least 30 min, and centrifuged for 10 min at 1,000 RCF to collect serum. Serum was stored at –80 °C until use. MaxiSorp Immuno plates (Thermo Fisher Scientific, Waltham, Massachusetts) were coated with 10 µg/ml recombinant MSP1_19_ overnight at 4 °C. Plates were blocked with PBS + 1% w/v BSA + 0.05% v/v Tween for 1 h at 37 °C. Serum was added to wells and incubated 2 h at RT. A gradient of ammonium thiocyanate starting at 4 M and ending at 0.15 M at a 1.6 dilution factor was added to the wells and incubated for 15 min at RT. Horseradish peroxidase–conjugated goat anti-mouse IgG (Jackson ImmunoResearch, West Grove, Pennsylvania) was added and incubated for 1 h at RT. Plates were developed with TMB substrate set (BioLegend, San Diego, California). Two moles H_2_SO_4_ was used to halt the reaction, and plates were read using a microplate reader with the absorbance read at 450 nm.

### ELISpot

To detect *P. yoelii* MSP1_19_-specific antibody-secreting cells, MultiScreen_HTS_ filter plates were coated with 10 µg/ml recombinant MSP1_19_ overnight at 4 °C. Bone marrow cells were collected and processed as described previously. Plates were washed with PBS and then blocked for 2 h at RT with RP-10 media. Dilutions of bone marrow cells were added to wells, and plates were incubated overnight at 37 °C. Plates were washed with PBS + 0.05% v/v Tween 20, and total MSP1_19_-specific IgM and IgG antibody-secreting cells were detected with horseradish peroxidase–conjugated goat anti-mouse IgM or IgG (Jackson ImmunoResearch, West Grove, Pennsylvania). Spots were developed using 3-amino-9-ethylcarbazole. Plates were read using an ImmunoSpot Analyzer and the ImmunoSpot 7.0.38.4 software (CTL, Cleveland, Ohio). The ImmunoSpot’s automated algorithm determined spots from background staining.

### Statistics

Statistical analysis was done using GraphPad Prism 10 (GraphPad Software, Boston, Massachusetts). Nonparametric testing (Mann–Whitney tests and Wilcoxon ranked-sum tests) were used owing to small population size and nonnormal distribution of the data.

## Results

### Gut microbiota impact the formation of immune memory cells at early but not late time points post–*P. yoelii* infection

We previously demonstrated that the composition of the gut microbiome influences the GC response to *P. yoelii* infection, and the ability of *P. yoelii*–immune mice to survive a lethal secondary challenge with *P. berghei* ANKA.^33^ We hypothesized that *P. yoelii* hyperparasitemia–resistant (resistant) mice were protected from *P. berghei* ANKA challenge by generating more MBCs and memory CD4 T cells than *P. yoelii* hyperparasitemia–susceptible (susceptible) mice in response to *P. yoelii* infection.

**Figure 1.**
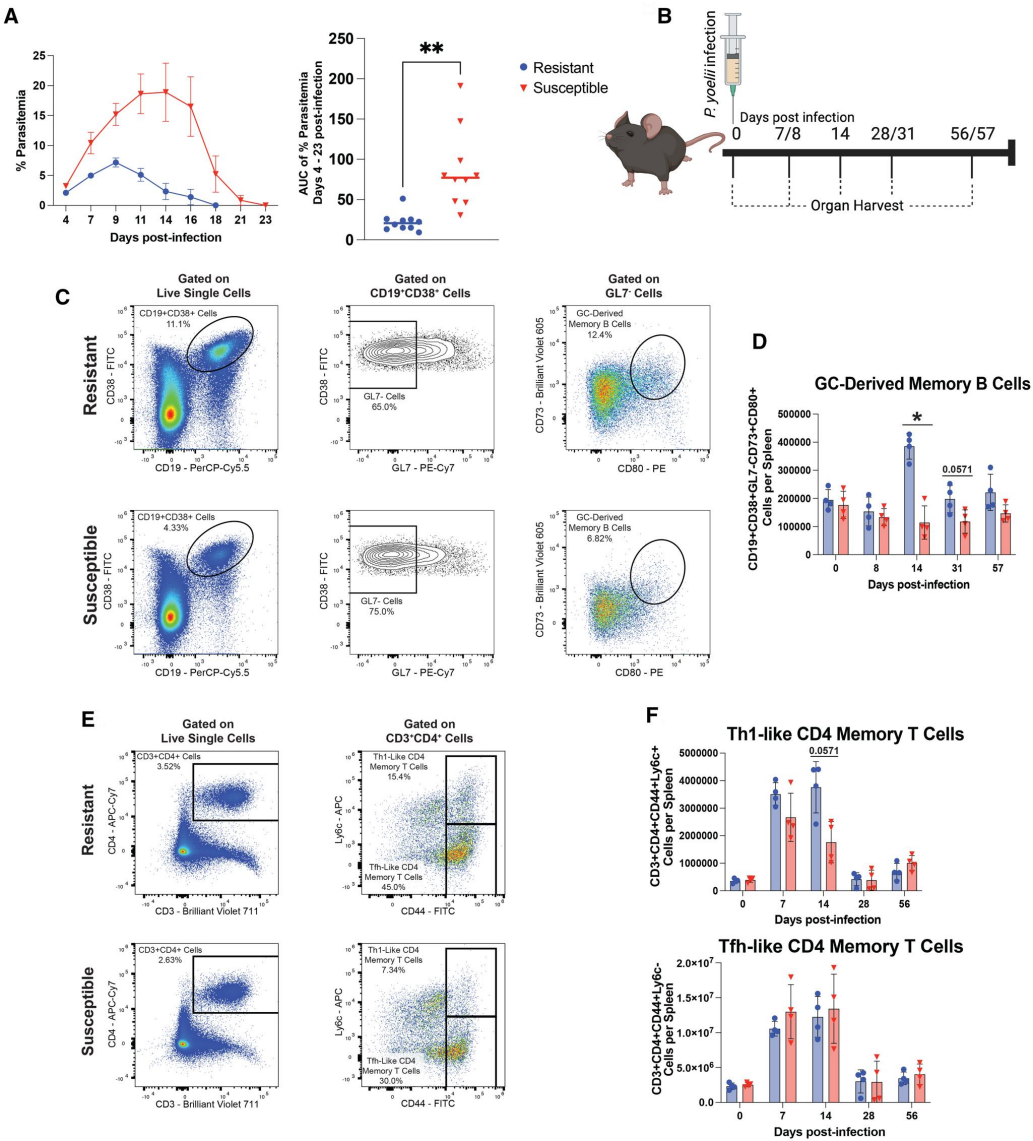
Resistant mice generate more immune memory cells during primary *P. yoelii* infection. C57BL/6 mice from Taconic Biosciences (Resistant) and Charles River Laboratories (Susceptible) were infected with *P. yoelii* 17XNL–infected red blood cells. All data in Fig. 1 are representative of 2 independent experiments. (A) Percentage of red blood cells infected with *P. yoelii* on indicated days p.i. (n = 4) (means ± SEM). Area under the parasitemia curve (AUC) was analyzed by Wilcoxon matched-pairs signed-rank test. (B) Experimental timeline schematic. Spleens were harvested on d 0, 7, 14, 28 or 31, and 56 or 67 p.i. with *P. yoelii*. Created with BioRender.com. (C and E) Representative FACS plots for GC-derived MBCs (CD19+CD38+GL7-CD73+CD80+), Th1-like CD4 memory T cells (CD3+CD4+Ly6c+CD44+), and Tfh-like CD memory T cells (CD3+CD4+Ly6c-CD44+) at d 14 p.i.. (D and F) Total number of GC-derived MBCs, Th1-like CD4 memory T cells, and Tfh-like CD memory T cells present in the spleen at indicated time points (n = 4) (means ± SEM) analyzed using Mann–Whitney test. *P < 0.05, **P < 0.01, ***P < 0.001, ****P < 0.0001.

Resistant and susceptible mice were infected with *P. yoelii,* and the parasite burden was evaluated. Susceptible mice had higher peak parasitemia and prolonged infection compared to resistant mice (Fig. 1A). Spleens were harvested between d 0 and 57 p.i. (Fig. 1B). Consistent with our hypothesis, resistant mice have more GC-derived MBCs during active *P. yoelii* infection compared to susceptible mice (Fig. 1C, D and Fig. S1A). However, the number of these cells present in the spleens of resistant and susceptible mice was similar on d 57 p.i., (Fig. 1D). Additionally, resistant mice trend towards having more Th1-like memory CD4 T cells present at d 14 p.i., but this difference subsides by d 28 (Fig. 1E, F and Fig. S1B). Furthermore, there are no differences in the number of Tfh-like memory CD4 T cells generated between resistant and susceptible mice (Fig. 1F). These results support our hypothesis that more MBCs and memory CD4 T cells are generated in resistant mice during *P. yoelii* infection. However, at more traditional memory time points (d 57 p.i.), these differences in memory B- and CD4 T-cell numbers are lost. This suggests that the total number of MBCs and memory CD4 T-cells present in the spleen are not contributing to the improved survival of resistant mice against *P. berghei* ANKA, but instead, there may be functional differences in these cells that contribute to the improved survival.

### Resistant and susceptible mice have similar numbers of bone marrow plasma cells and circulating *P. yoelii*–specific antibodies following *P. yoelii* infection

The blood-stage of *Plasmodium* infection is primarily cleared in an antibody-dependent manner. For this reason, we next characterized the PC response generated in response to *P. yoelii* infection. We followed the same experimental timeline outlined in Fig. 1B and harvested bone marrow at the indicated time points. While PCs are generated within the GC in secondary lymphoid organs, they travel to the bone marrow to survive as long-lived PCs (LLPCs), where they continuously secrete antigen-specific antibodies to protect against reinfection.^35^ For this reason, we focused our studies on bone marrow–resident PCs. We hypothesized that resistant mice would see greater accumulation of LLPCs in the bone marrow following *P. yoelii* infection.

**Figure 2.**
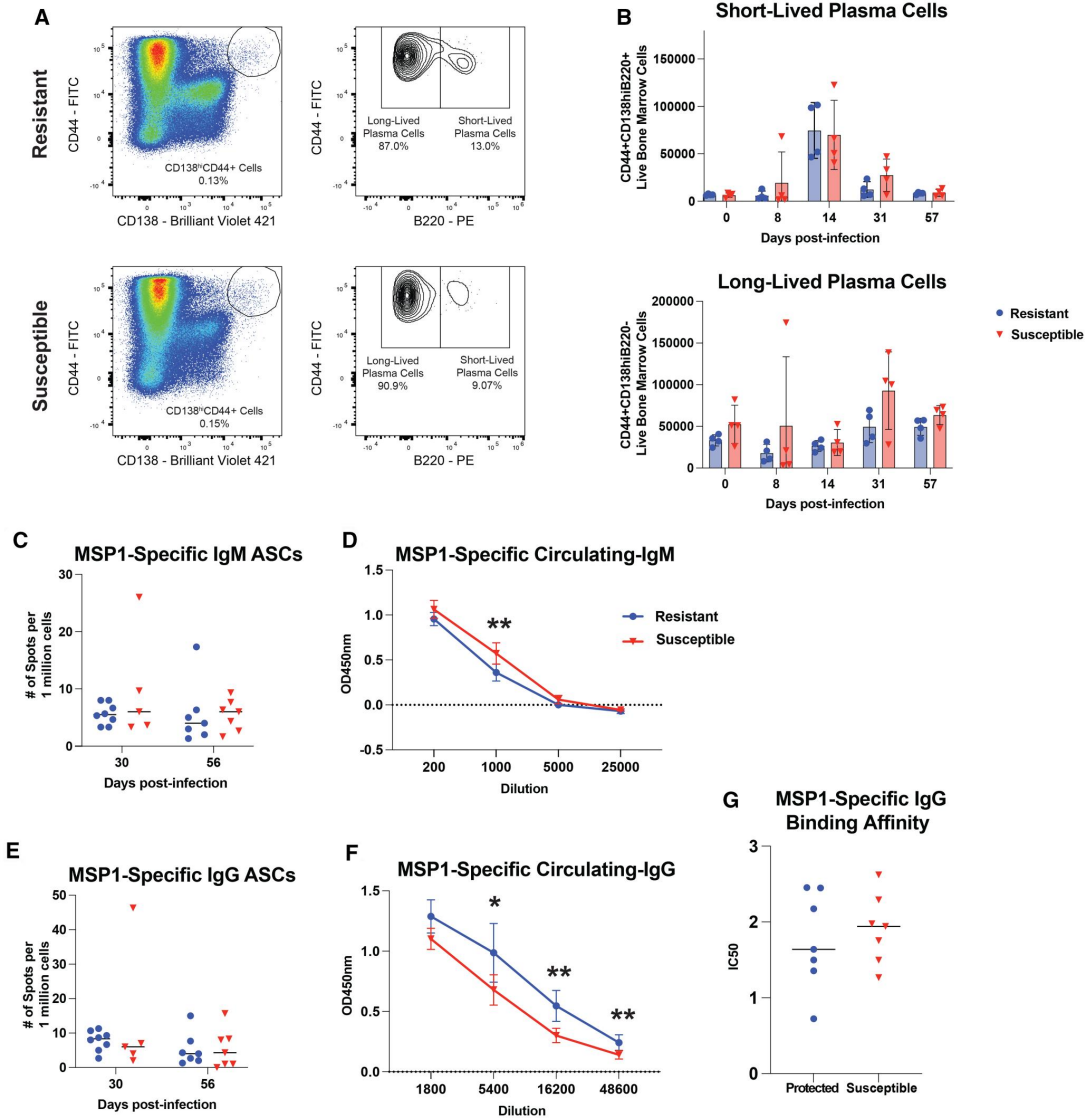
Resistant and susceptible mice have similar accumulation of plasma cells in the bone marrow following *P. yoelii* infection. (A) Representative FACS plots for SLPCs (CD138hiCD44+B220+) and LLPCs (CD138hiCD44+B220–) at d 57 p.i. (B) Total number of SLPCs and LLPCs present in the bone marrow at indicated time points (n = 4) (means ± SEM) analyzed using unpaired *t* test. Data are representative of 2 independent experiments. (C and E) ELISpot analysis of total number of MSP1_19_-specific IgM- and IgG-secreting cells present in the bone marrow (n = 7 or 8) (means ± SEM). Data were collected in triplicates per sample and the average value was taken and plotted. Data are pooled from 2 independent experiments and analyzed using Mann–Whitney test. (D) Sera were collected at d 56 p.i., diluted as indicated and reacted against MSP1_19_-coated plates to detect IgM antibodies by ELISA (n = 7 or 8). Data are pooled from 2 independent experiments and analyzed using Mann–Whitney test. (F) Sera were collected at d 56 p.i., diluted as indicated, and reacted against MSP1_19_-coated plates to detect IgG antibodies by ELISA (n = 7 or 8). Data are pooled from 2 independent experiments and analyzed using Mann–Whitney test. (G) IC_50_ of ammonium thiocyanate needed to remove IgG binding to MSP119. Sera were collected on d 56 p.i. with *P. yoelii*, and the amount of MSP1_19_-specific IgG was quantified (n = 7 or 8). Equal amounts of MSP1_19_-specific IgG were incubated with increasing concentrations of ammonium thiocyanate. The percentage of binding of ammonium thiocyanate treated samples was compared with untreated samples to calculate IC_50_ for each individual. Data are pooled from 2 independent experiments and analyzed using Mann–Whitney test. *P < 0.05, **P < 0.01, ***P < 0.001, ****P < 0.0001.

B220 is used to distinguish between short-lived PCs (SLPCs) and LLPCs, with LLPCs having lost B220 expression.^36^ We characterized SLPCs as being CD138hiCD44+B220+ and LLPCs as being CD138hiCD44+B220– cells (Fig. 2A). Following *P. yoelii* infection, there was no difference in the number of SLPCs or LLPCs accumulating in the bone marrow of resistant and susceptible mice (Fig. 2B and Fig. S2A). As this phenotypic assessment captures all PCs, including both *P. yoelii*–specific and environment-induced PCs, we next aimed to determine if there were differences in the number of *P. yoelii*–specific PCs accumulating in the bone marrow. We accomplished this by using ELISpot to identify merozoite surface protein 1_1-19_ (MSP1_19_) antibody-secreting cells. MSP1_19_ is an immunodominant protein present on the surface of *P. yoelii* merozoites.^37^ Again, we found there were no differences in the number of MSP1_19_-specific IgM- or IgG-secreting cells in the bone marrow of resistant and susceptible mice (Fig. 2C, E).

Because there were no differences in the number of PCs present in the bone marrow, we hypothesized that the PCs in the bone marrow of resistant mice have a greater functional capacity than those found in susceptible mice. Using ELISpot, we identified that MSP1_19_-specific IgM- or IgG-secreting cells in the bone marrow of resistant and susceptible mice secrete comparable amounts of MSP1_19_-specific antibodies (Fig. S2B. C). However, susceptible mice had a greater amount of MSP1_19_-specific circulating IgM antibody titers at d 56 p.i., while resistant mice had more MSP1_19_-specific IgG (Fig. 2D, F). Increased *P. yoelii*–specific total IgG antibody titers were not attributed to either MSP1_19_-specific IgG1 or IgG2b antibodies (Fig. S2D). In spite of the slight increase in MSP1_19_-specific IgG in resistant mice, the affinity of MSP1_19_-specific IgG was similar between the 2 groups (Fig. 2G). Similar observations were also evident following the evaluation of the bulk *P. yoelii*–specific antibody response using infected red blood cell protein lysate (Fig. S2E). These results indicate that the composition of the gut microbiome does not influence the ability of LLPCs to accumulate in the bone marrow following *P. yoelii* infection or their long-term ability to secrete *P. yoelii*–specific antibodies post–parasite clearance.

### Resistant mice generate a more robust secondary GC B-cell response to *P. berghei* ANKA

**Figure 3.**
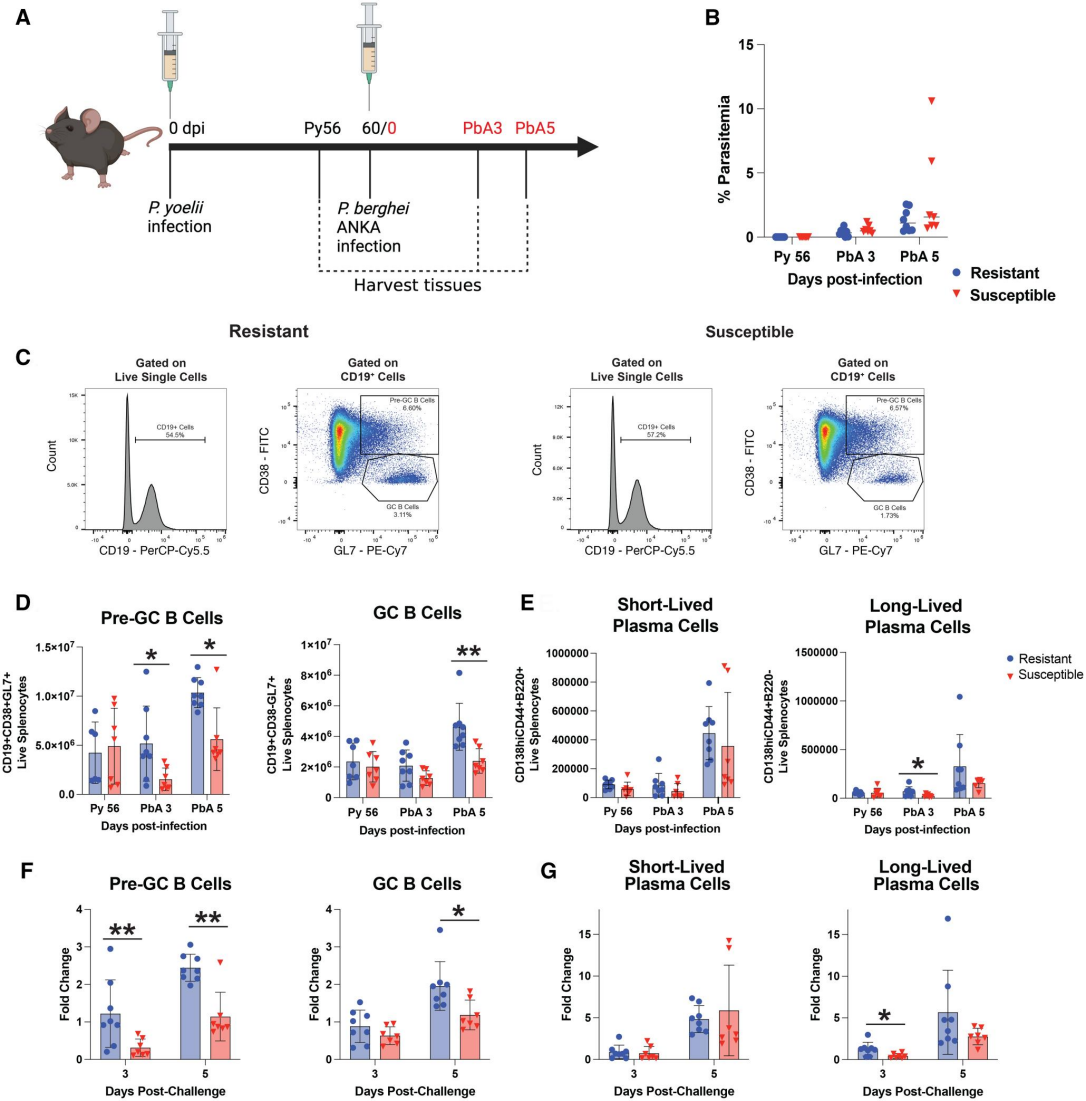
Resistant mice generate a more robust secondary GC B-cell response to *P. berghei* ANKA. (A) Schematic outlining experimental design. Mice were infected with *P. yoelii*, and 60-d–p.i. mice were challenged with *P. berghei* ANKA. Spleens were harvested at d 56 post–*P. yoelii* infection (Py 56), and at d 3 and 5 post–*P. berghei* ANKA challenge (PbA 3 and 5). Created with BioRender.com. (B) Percent parasitemia of mice on day of spleen harvest (n = 7 or 8). Data (mean ± SEM) are pooled from 2 independent experiments and analyzed using Mann–Whitney test. (C) Representative FACS plots for pre-GC B cells (CD19+CD38+GL7+) and GC B cells (CD19+CD38-GL7+) at d 5 p.c. (D and E) Total number of pre-GC B cells, GC B cells, SLPCs (CD138hiCD44+B220+), and LLPCs (CD138hiCD44+B220–) present in the spleen at indicated time points (n = 7 or 8) (means ± SEM) analyzed using Mann–Whitney test. Data are pooled from 2 independent experiments. (F and G) Fold change of pre-GC B cells, GC B cells, SLPCs, and LLPCs at d 3 and 5 post–*P. berghei* ANKA challenge compared to d 56 post–*P. yoelii* infection (n = 7 or 8). Calculated by dividing cell numbers at d 3 and 5 p.c. by the mean number of cells present at d 56 post–*P. yoelii* infection. Data (means ± SEM) analyzed using Mann–Whitney test. Data are pooled from 2 independent experiments. *P < 0.05, **P < 0.01, ***P < 0.001, ****P < 0.0001.

Similar to our previous study, *P. yoelii*–immune resistant mice exhibited greater protection against ECM following *P. berghei* ANKA challenge compared to *P. yoelii*–immune susceptible mice (Fig. S3A–C). Humane endpoints were determined on the basis of neurocognitive decline and progression towards coma/seizure. Because the humoral immune system is crucial for clearing blood-stage *Plasmodium* infection, we next assessed GC responses following *P. berghei* challenge. These GC responses could be seeded by a mixture of *P. yoelii*–specific MBCs and memory CD4 T cells along with *P. berghei*–specific naïve B cells and CD4 T cells. We hypothesized that *P. yoelii*–immune resistant mice would generate a more robust GC-associated B-cell response than *P. yoelii*–immune susceptible mice. To test this, we harvested spleens from *P. yoelii*–immune mice 4 d prior to *P. berghei* ANKA challenge (Py 56), and at d 3 and 5 postchallenge (p.c.) (PbA 3 and PbA 5) (Fig. 3A). Both resistant and susceptible mice had similar parasite burdens at the time of spleen harvest (Fig. 3B). In line with our hypothesis, resistant mice produced more pre-GC B cells and GC B cells in response to *P. berghei* ANKA secondary challenge (Fig. 3C, D). However, there was little to no difference in the number of SLPCs, LLPCs, or plasmablasts produced in the spleen during *P. berghei* ANKA secondary challenge (Fig. 3E, Fig. S4B). Additionally, there were no differences in the number of GC-derived MBCs present in the spleen p.c. (Fig. S4D). We next investigated differences in fold change of these populations from Py d 56 p.i. Resistant mice had a statistically greater fold change difference in pre-GC B cells, GC B cells, and LLPCs (Fig. 3F, G). These results suggest that resistant mice generate a stronger secondary humoral immune response to *P. berghei* ANKA, potentially contributing to their heightened survival against *P. berghei* ANKA–induced ECM.

### Circulating antibody levels do not directly correlate to protection from *P. berghei* ANKA secondary challenge

**Figure 4.**
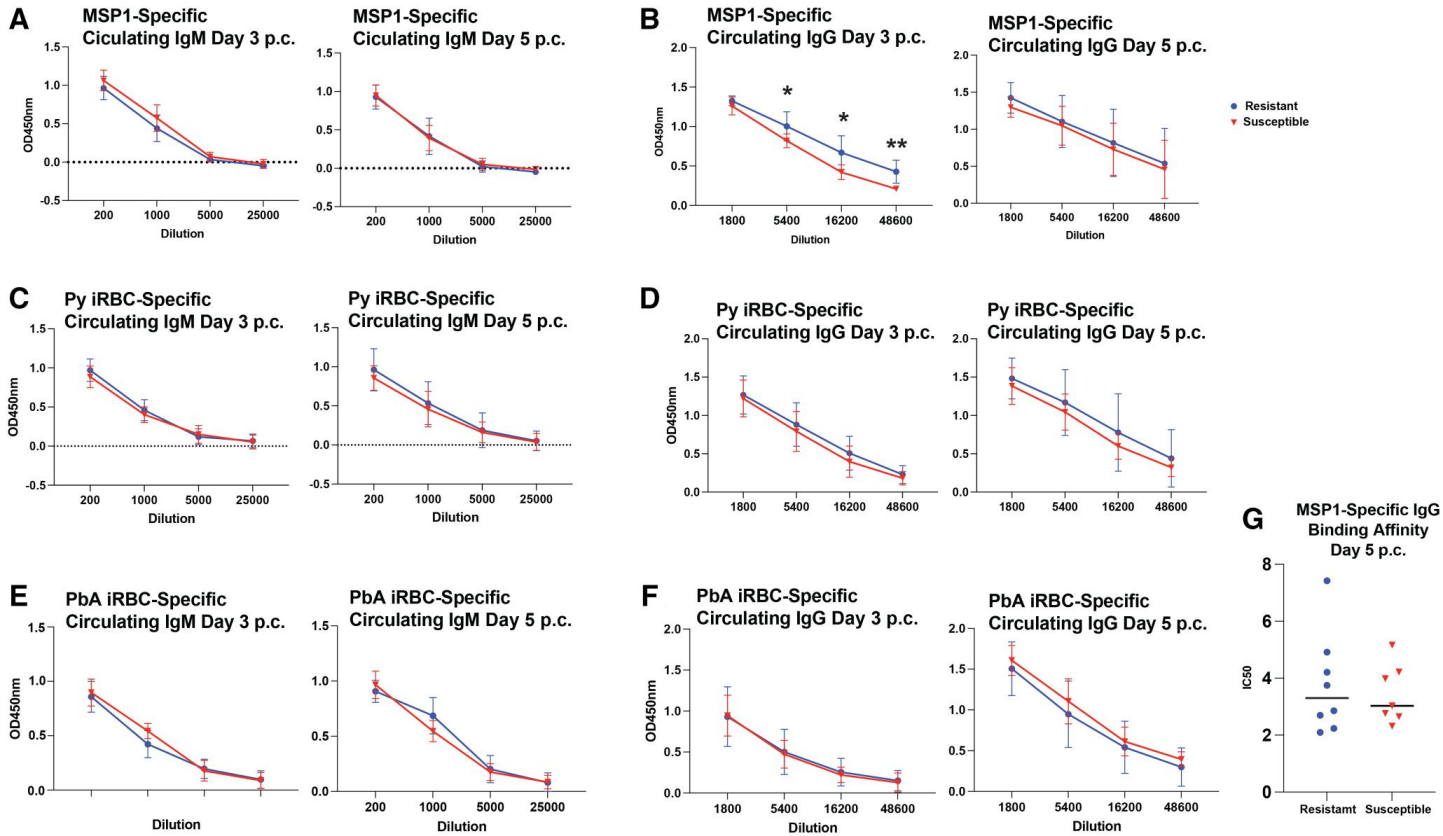
Similar circulating antibody titers in resistant and susceptible mice following *P. berghei* ANKA secondary challenge. (A and B) Sera were collected at d 3 and 5 p.c., diluted as indicated, and reacted against MSP1_19_-coated plates to detect IgM and IgG antibodies by ELISA (n = 7 or 8). Data are pooled from 2 independent experiments and analyzed using Mann–Whitney test. (C and D) Sera were collected at d 3 and 5 post–*P. berghei* ANKA challenge, diluted, and reacted against plates coated with proteins from lysed *P. yoelii* infected red blood cells to detect IgM and IgG antibodies by ELISA (n = 7 or 8). Data (means ± SEM) are pooled from 2 independent experiments and analyzed using Mann–Whitney test. (E and F) Sera were collected at d 3 and 5 post–*P. berghei* ANKA challenge, diluted, and reacted against plates coated with proteins from lysed P. yoelii infected red blood cells to detect IgM and IgG antibodies by ELISA (n = 7 or 8). Data (means ± SEM) are pooled from 2 independent experiments and analyzed using Mann–Whitney test. (G) IC_50_ of ammonium thiocyanate needed to remove IgG binding to MSP1_19_. Sera were collected at d 3 and 5 p.c. with *P. berghei* ANKA, and the amount of MSP1_19_-specific IgG was quantified (n = 7 or 8). Equal amounts of MSP1_19_-specific IgG were incubated with increasing concentrations of ammonium thiocyanate. The percentage of binding of ammonium thiocyanate treated samples was compared with untreated samples to calculate IC_50_ for each individual sample. Data are pooled from 2 independent experiments and analyzed using Mann–Whitney test. *P < 0.05, **P < 0.01, ***P < 0.001, ****P < 0.0001.

Owing to the more robust GC response generated by *P. yoelii*–immune resistant mice in response to *P. berghei* ANKA challenge, we hypothesized that *P. yoelii*–immune resistant mice have increased titers of *P. yoelii* MSP1_19_-specific circulating antibodies p.c. that confer cross-reactive protection against *P. berghei* ANKA–induced ECM. To test this, we collected sera from *P. berghei* ANKA-infected mice on d 3 and 5 p.c. prior to ECM onset on d 6 p.c. We found that both resistant and susceptible mice have comparable amounts of *P. yoelii* MSP1_19_-specific circulating IgM at both d 3 and 5 p.c. (Fig. 4A). In contrast, we found that resistant mice had more *P. yoelii* MSP1_19_-specific IgG at d 3 p.c., but this difference was lost by d 5 p.c. (Fig. 4B). This was consistent with a similar affinity of *P. yoelii* MSP1_19_-specific IgG between *P. yoelii*–immune resistant and susceptible mice 5 d p.c. (Fig. 4G). Assessment of individual IgG isotypes demonstrated no differences in *P. yoelii* MSP1_19_-specific IgG1 or IgG2b circulating antibodies at d 5 p.c. (Fig. S5A, B).

The total antibody response to *P. yoelii* was also evaluated in *P. berghei* ANKA–challenged mice using parasite lysate from *P. yoelii*–infected red blood cells. Consistent with the minimal differences in *P. yoelii* MSP1_19_-specific antibodies in *P. yoelii*–immune resistant and susceptible mice challenged with *P. berghei*, there were no differences in antibody titers against *P. yoelii* parasite lysate in either group of mice on d 3 or 5 p.c. (Fig. 4C, D and Fig. S5C, D). Finally, we assessed *P. berghei* ANKA–specific antibody titers, including both *P. berghei*–induced antibody responses and *P. yoelii*–induced antibodies that were cross-reactive with *P. berghei* ANKA. Despite the increased number of GC B cells in *P. yoelii*–immune resistant mice, there were no differences in circulating *P. berghei* ANKA–infected red blood cell lysate IgM- and IgG-specific antibody titers (Fig. 4E, F), which is consistent with the similar *P. berghei* ANKA parasite burden in both groups of mice at these time points (Fig. 3B). This suggests alternative components of the host immune response confer protection against ECM.

### Resistant mice generate a more robust secondary GC T-cell and regulatory T-cell response to *P. berghei* ANKA

**Figure 5.**
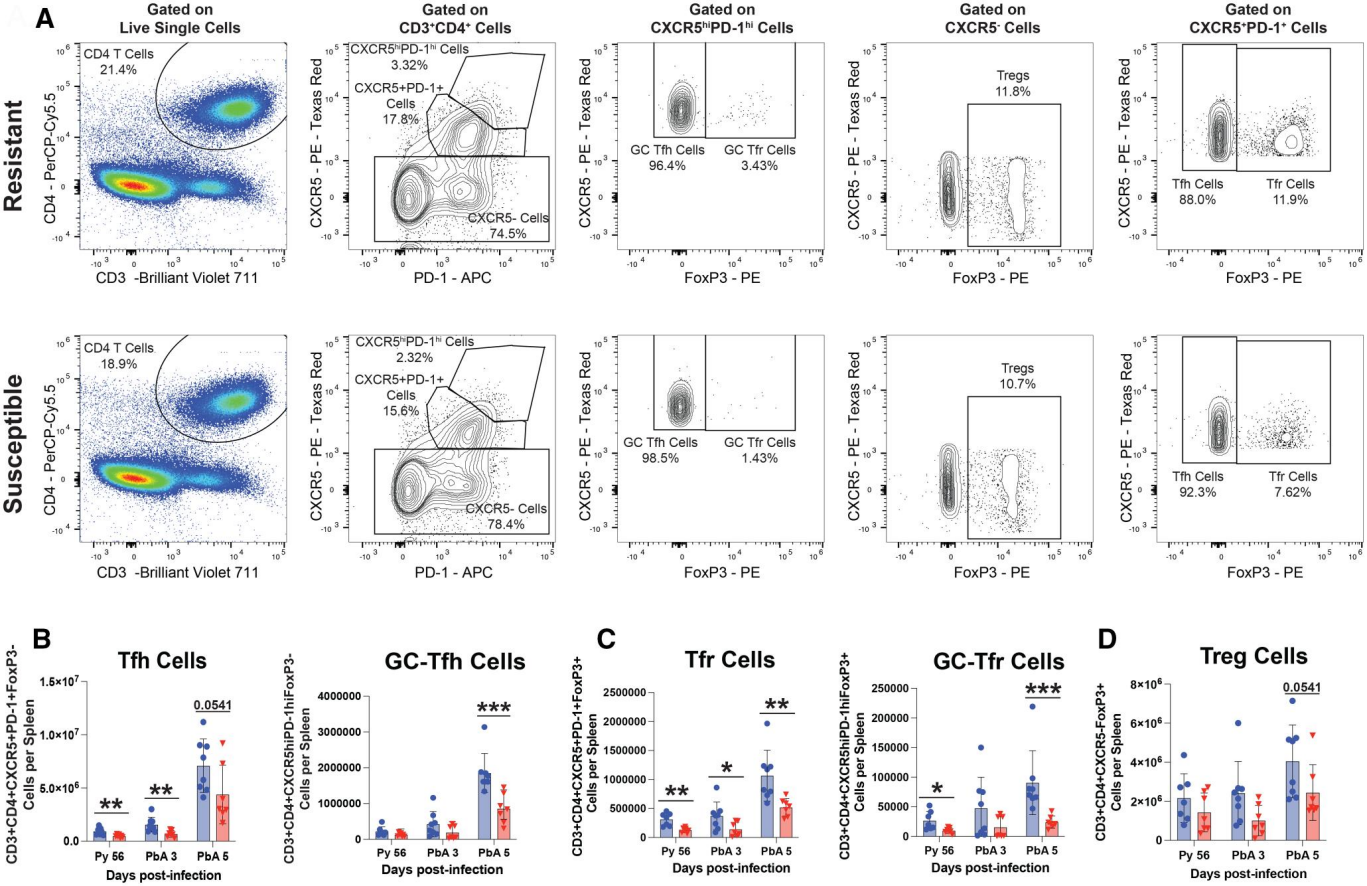
Resistant mice generate a more robust secondary GC T-cell and regulatory T-cell response to *P. berghei* ANKA. (A) Representative FACS plots for Tfh cells (CD3+CD4+CXCR5+PD-1+FoxP3–), Tfr cells (CD3+CD4+CXCR5+PD-1+FoxP3+), GC-Tfh cells (CD3+CD4+CXCR5hiPD-1hiFoxP3–), GC-Tfr cells (CD3+CD4+CXCR5hiPD-1hiFoxP3+), and Treg cells (CD3+CD4+CXCR5–FoxP3+) at d 5 p.c. (B–D) Total number of Tfh cells, GC-Tfh cells, Tfr cells, GC-Tfr cells, and Treg cells present in the spleen at indicated time points (n = 7 or 8) (means ± SEM) analyzed using Mann–Whitney test. Data are pooled from 2 independent experiments. *P < 0.05, **P < 0.01, ***P < 0.001, ****P < 0.0001.

We also investigated the GC-associated T-cell response to *P. berghei* ANKA secondary challenge. We hypothesized that resistant mice would generate a more robust effector T-cell response than susceptible mice. To test this, we harvested spleens from *P. yoelii*–immune mice 4 d prior to *P. berghei* ANKA challenge (Py 56), and at d 3 and 5 p.c (PbA 3 and PbA 5) (Fig. 3A). We observed that resistant mice generated more Tfh and GC-Tfh cells in response to *P. berghei* ANKA challenge (Fig. 5A, B), and had more Th1-like CD4 memory T cells or Tfh-like CD4 memory T cells present in the spleen p.c. (Fig. S6A, B). This is consistent with the resistant mice generating an overall stronger secondary GC response when challenged with *P. berghei* ANKA. However, our data suggest that protection from ECM in *P. yoelii*–immune mice is unlikely to be attributed to better GC responses.

Curiously, we observed resistant mice also produced more Tfr and GC-Tfr cells than susceptible mice (Fig. 5C). Additionally, we observed a trend towards increased numbers of Tregs in *P. yoelii*–immune resistant mice (1.66-fold difference: *P* = 0.0541) following *P. berghei* ANKA challenge (Fig. 5D). These results demonstrate that resistant mice generate both more effector and regulatory T cells in response to *P. berghei* ANKA secondary challenge.

## Limitations

During these investigations, the gut microbiome composition of the resistant mice from Taconic Biosciences shifted. This change has rendered the previously resistant mice susceptible to *P. yoelii* hyperparasitemia, and they no longer protected from *P. berghei* ANKA–associated mortality. For this reason, we are unable to further investigate what components of immune memory protect against *P. berghei* ANKA secondary challenge in this model.

## Discussion

We have previously established that the composition of the gut microbiome impacts the primary immune response to *P. yoelii* by influencing the GC response and the formation of *P. yoelii*–specific antibodies.^33^ Additionally, we have determined that *P. yoelii*–resistant mice are more likely to survive when reinfected with a lethal *P. berghei* ANKA secondary challenge.^33^ This current study has provided insight into the influence of the gut microbiome on the formation of immune memory cells and the recall response to a secondary *Plasmodium* challenge. These data show that the composition of the gut microbiome impacts the formation of memory B- and T-cell populations during primary *P. yoelii* infection but not accumulation p.i. This suggests that the gut microbiome may instead impact the ability of these cells to respond to reinfection. This is demonstrated by resistant mice mounting a more robust GC response to secondary challenge with *P. berghei* ANKA. However, *P. yoelii*–resistant mice also generated more regulatory T cells (Tfrs, GC-Tfrs, and a trend towards more Tregs) during the secondary *P. berghei* ANKA challenge. These cells may contribute to protection against *P. berghei* ANKA–induced ECM by dampening the overall immune response and preventing the migration of T cells to the brain.

Previous work has demonstrated that naturally acquired immunity to *Plasmodium* develops over years of repeated exposure and the acquisition of a broad array of *Plasmodium*-specific antibodies.^38–40^ These antibodies are primarily generated by plasma cells, which reside in the bone marrow and continuously secrete antigen-specific antibodies for years after exposure.^35^ Work from other groups had established that while plasma cells do form in response to *P. yoelii* infection, their generation is impaired.^41^ We unfortunately did not look at the formation of plasma cells in the spleen during primary *P. yoelii* infection, but our results here demonstrate that the gut microbiome does not impact the accumulation of plasma cells in the bone marrow following *P. yoelii* infection. We can infer from these results that the gut microbiome is not impacting the plasma cell niche in the bone marrow, therefore not altering the ability of plasma cells to engraft in the bone marrow following infection, but more experimentation would need to be done to confirm this. Additionally, the gut microbiome does not appear to influence the functionality of these plasma cells, with resistant and susceptible mice generating similar amounts of *Plasmodium-*specific circulating antibody titers and similar binding affinity to *P. yoelii* MSP1_19_. Furthermore, resistant and susceptible mice have a similar quantity of *Plasmodium-*specific circulating antibody titers immediately before the onset of ECM, indicating that circulating antibody levels do not correlate with protection from *P. berghei* ANKA–induced ECM. This suggests that alternate components of the host immune response confer protection against ECM.

Cerebral malaria is the most devastating complication of severe malaria, and even with treatment, the mortality rate of cerebral malaria is still around 15% to 20%.^7^^,^^8^ While hypotheses exist, the underlying cause of cerebral malaria in humans is unknown. In mice, ECM is caused by the infiltration of CD8 T cells to the brain, causing the breakdown of the blood–brain barrier and inducing brain swelling.^42–44^ There is evidence that T-cell infiltration may also play a role in human cerebral malaria.^45^ While Tregs are known to regulate the function and development of CD8 T cells, and in diabetes, Tregs can modulate the migration of CD8 T cells into the pancreatic islets,^46–48^ they do not appear to play much of a role regulating the CD8+ T-cell–mediated breakdown of the blood–brain barrier in ECM.^49^ However, the overall role of Tregs in ECM is much more unclear, with some groups reporting that Tregs help prevent ECM-associated immunopathologies,^49–51^ and others reporting that Tregs contribute to pathology.^52^^,^^53^ Intriguingly, one group found that BALB/c mice, which are protected from *P. berghei* ANKA–induced ECM, have higher numbers of Tregs during *P. berghei* ANKA infection than C57BL/6 mice.^51^ When Tregs were depleted from these mice, they showed worsened symptoms of ECM, although they ultimately did not succumb to ECM.^51^ They also demonstrated that Tregs modulate the functionality of CD4+ T cells and dampen the Th1 response, which is associated with worsened outcomes to ECM.^51^ Using other lethal strains of *Plasmodium* infections, some groups have found that depleting Tregs improves survival and parasite clearance,^54^^,^^55^ while others show no difference or worsened survival.^56^^,^^57^ Depleting Tregs during *Plasmodium* infection has been shown to increase IFN-γ levels, which negatively impacts GCs.^41^^,^^57^ As with many other immune responses to *Plasmodium*, it is likely that at a baseline Tregs are important in preventing immunopathology, but too strong of a regulatory response can increase parasite-associated pathologies. We hypothesize that the Treg cells in our resistant mice behave similarly to how they have been previously reported in BALB/c mice^51^—contributing to protection from symptoms but not necessary for survival.

While Tfrs do not regulate CD8 T cells, they are implicated in modulating the GC response by regulating Tfh cells and B cells.^58^ In other studies, it has been demonstrated that Tfrs are necessary for Tfh to GC B-cell interactions, and a loss of Tfrs can cause altered antibody responses.^59^^,^^60^ Additionally, Tfrs have been shown to regulate the formation of plasma cells, and depleting Tfrs increased the number of plasma cells present.^59^^,^^61^ To our knowledge, no studies have observed if plasma cells are present in the brains of mice succumbing to ECM. However, plasma cells are known to contribute to brain swelling in other diseases, such as encephalitis.^62^^,^^63^ If plasma cells contribute to ECM pathology, it is possible that Tfrs in resistant mice contribute to survival from ECM by strengthening the GC and by regulating the plasma cell response. Future research will need to be conducted to elucidate the role of Tregs and Tfrs in protection from ECM. Further understanding in this area could lead to potential treatments and therapies that modulate Treg function to protect individuals from cerebral malaria and could also be used to guide future vaccine development.

Currently, there are 2 vaccines authorized for use in humans against *Plasmodium*. While both vaccines generate good protection initially (60% to 75% one-year postadministration), this protection wanes after the end of the vaccination series.^1^^,^^2^^,^^4^^,^^5^ For this reason, there is still much we need to do to develop strong, long-lasting vaccines against *Plasmodium* in humans. We have demonstrated that the gut microbiome influences the effector and memory immune responses against *Plasmodium*. Additionally, this work is important in understanding how the gut microbiome may influence the formation of vaccine-induced immune memory, and the ability of these immune memory cells to respond upon infection. Previous studies have shown that the composition of the gut microbiome can influence the efficacy of vaccines.^64–67^ Additionally, it has been found that the composition of the gut microbiome earlier in life can correlate with the strength of immune responses later on, including vaccine-induced immune responses.^68^ This work could support a line of research to investigate what consortium of microbes in the gut may be the most beneficial for maintaining a population of functional immune memory cells following vaccination. Gaining a better understanding of this link between the gut microbiome and the immune system is crucial in the ongoing work to develop a long-lasting, effective vaccine for *Plasmodium*.

There are many advantages to using mouse models in immunology research, but there are also important limitations. For example, bacteria colonize and expand differently in the intestinal tract of mice compared to humans, meaning that the gut microbiome we observe in mice is not entirely translatable to humans. While mouse studies offer a good starting point, human samples will need to be utilized in future experimentation to fully elucidate the role of the gut microbiome in influencing the immune memory response to *Plasmodium.*

**Figure 6.**
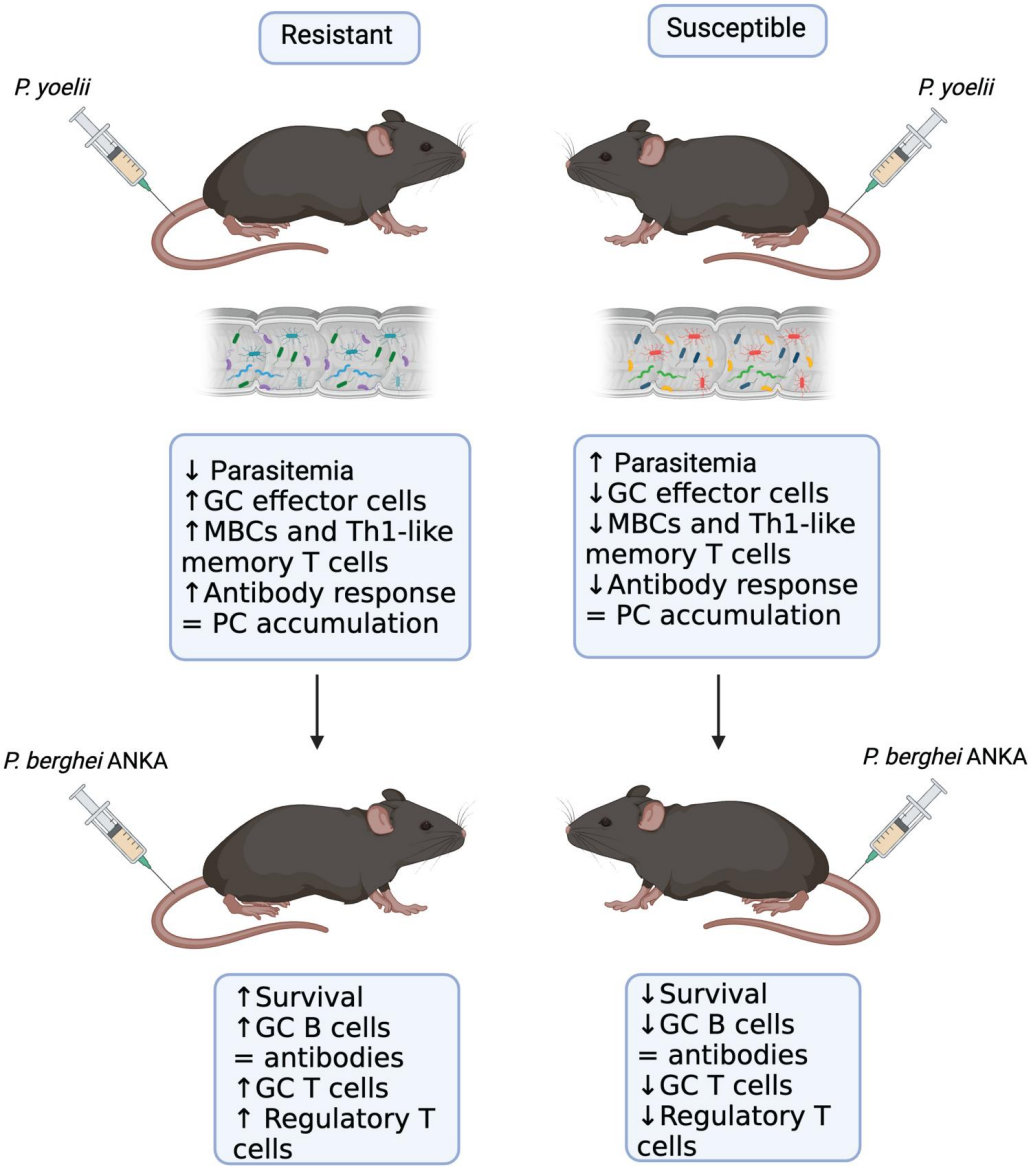
The composition of the gut microbiome impacts the formation and functionality of *P. yoelii*–induced immune memory cells. Graphical abstract summarizing the key findings of this article. Created with BioRender.com.

This study demonstrates that the gut microbiome influences the functionality of immune memory cells generated in response to *P. yoelii* infection in mice, therefore impacting the recall GC response to *P. berghei* ANKA secondary challenge (Fig. 6). This work supports the notion that the gut microbiome can be manipulated to increase the rate at which one develops clinical immunity to malaria.

## Supplementary Material

vlaf009_Supplementary_Data

## Data Availability

The data underlying this article will be shared on reasonable request to the corresponding author.
