# Supplementary material for: Microbiome-mediated modulation of immune memory to P. yoelii affects the resistance to secondary cerebral malaria challenge
Source: Immunohorizons. 2025 Mar 28;9(5):vlaf009. doi: 10.1093/immhor/vlaf009 (PMC12086675; doi:10.1093/immhor/vlaf009)
Supplement: vlaf009_Supplementary_Data [file vlaf009_Supplementary_Data.pdf]

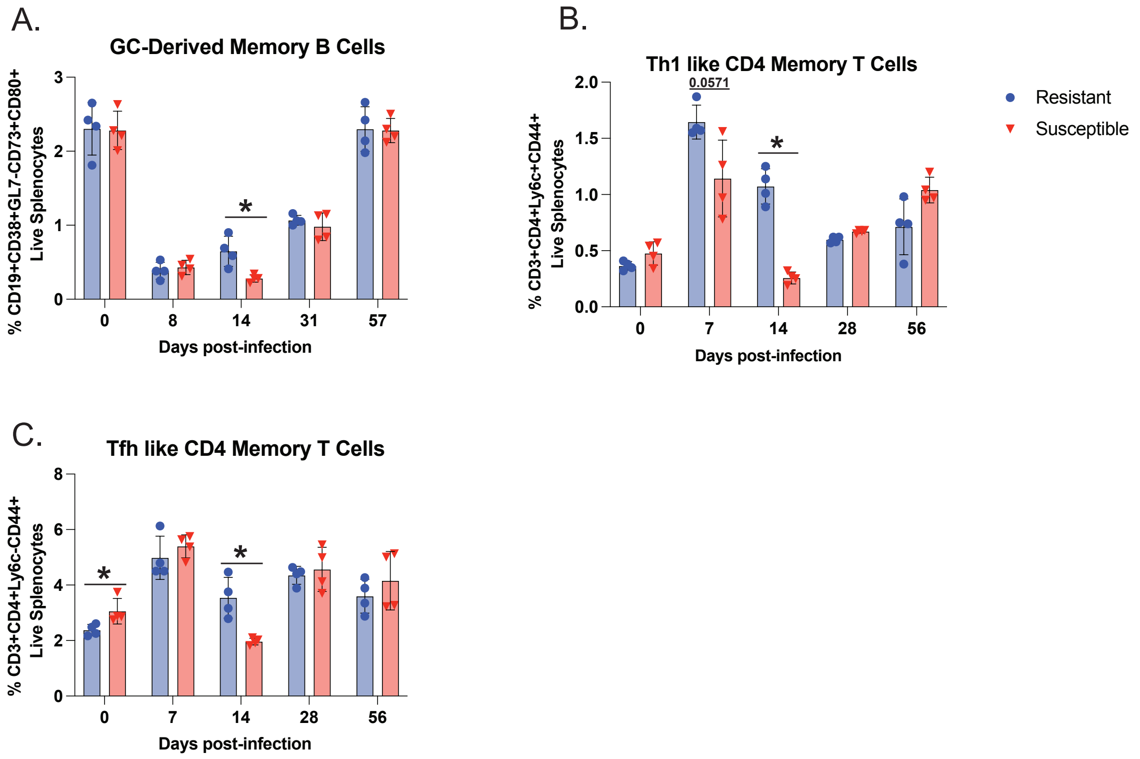

Supplemental Figure 1: Resistant mice have a greater frequency of MBCs and memory T cells at day 14 post infection with P. yoelii

(A, B, and C) Percentage of GC-Derived MBCs, Th1 like CD4 memory T cells, and Tfh like CD memory T cells present in the spleen at indicated time points post infection with *P. yoelii* (n=4) (means +/- SEM) analyzed using Mann-Whitney test. Data are representative of two independent experiments.

*p<0.05, **p<0.01, ***p<0.001, ****p<0.0001

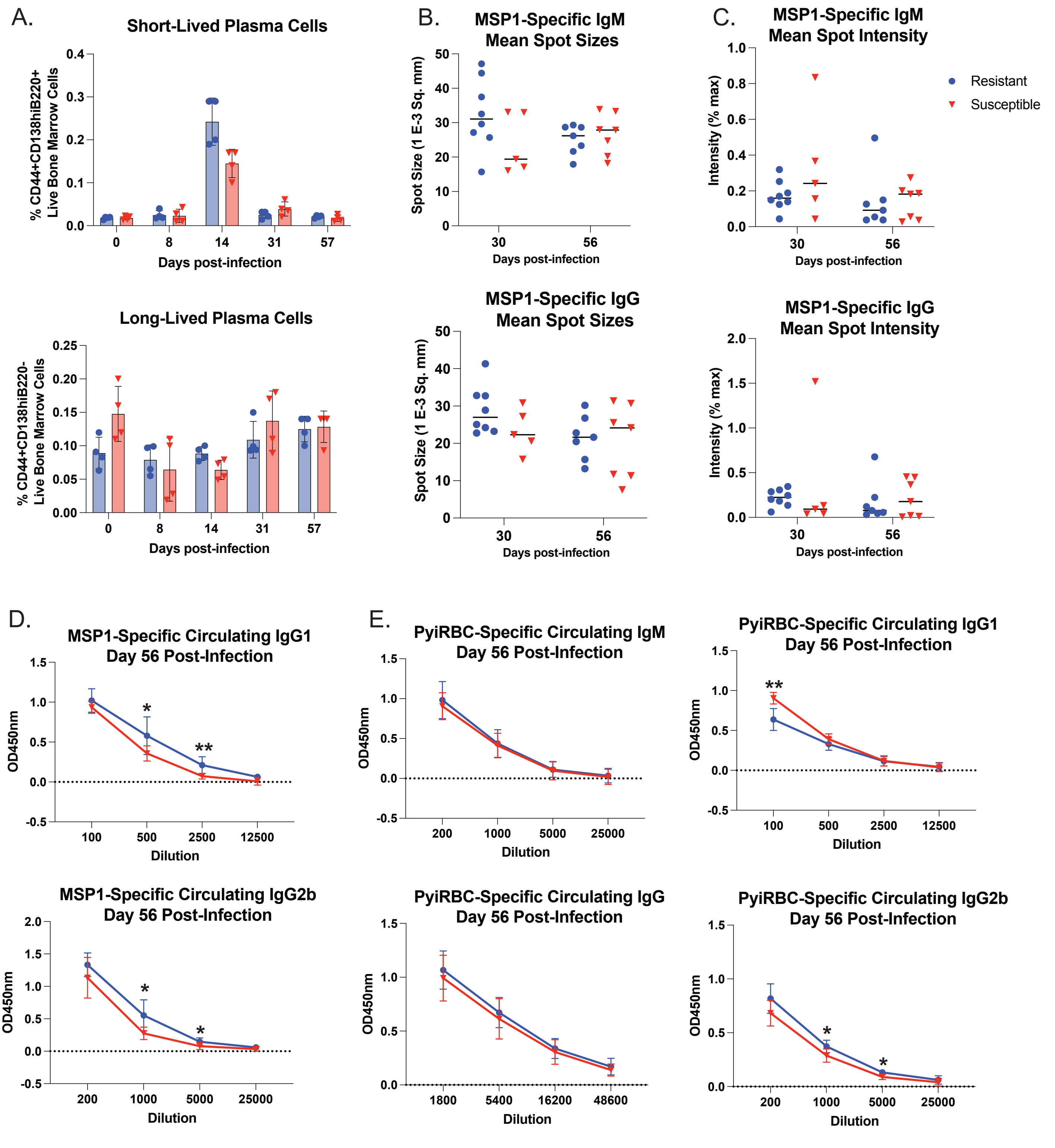

Supplemental Figure 2: Resistant and susceptible mice generate a similar plasma cell response to *P. yoelii* infection
(A) Percentage of SLPCs and LLPCs present in the bone marrow at indicated time points post infection with *P. yoelii* (n=4) (means +/- SEM) analyzed using unpaired t test. Data are representative of two independent experiments.
(B and C) ELISpot analysis of size of spots, and darkness of spots generated by $MSP1_{19}$ specific IgM and IgG secreting cells present in the bone marrow post infection with *P. yoelii* (n=7 or 8) (means +/- SEM). Data was collected in triplicates per sample and the average value was taken and plotted. Data (means +/- SEM) are cumulative of two experiments and analyzed using Mann-Whitney test.
(D) Sera was collected at day 56 post infection, diluted, and reacted against $MSP1_{19}$ coated plates to detect IgG1 and IgG2b antibodies by ELISA (n=7 or 8). Data (means +/- SEM) are cumulative of two experiments and analyzed using Mann-Whitney test.
(E) Sera was collected at day 56 post infection, diluted, and reacted against lysed *P. yoelii* infected red blood cell protein-coated plates to detect IgGM, IgG, IgG1 and IgG2b antibodies by ELISA (n=7 or 8). Data (means +/- SEM) are cumulative of two experiments and analyzed using Mann-Whitney test.
*p<0.05, **p<0.01, ***p<0.001, ****p<0.0001

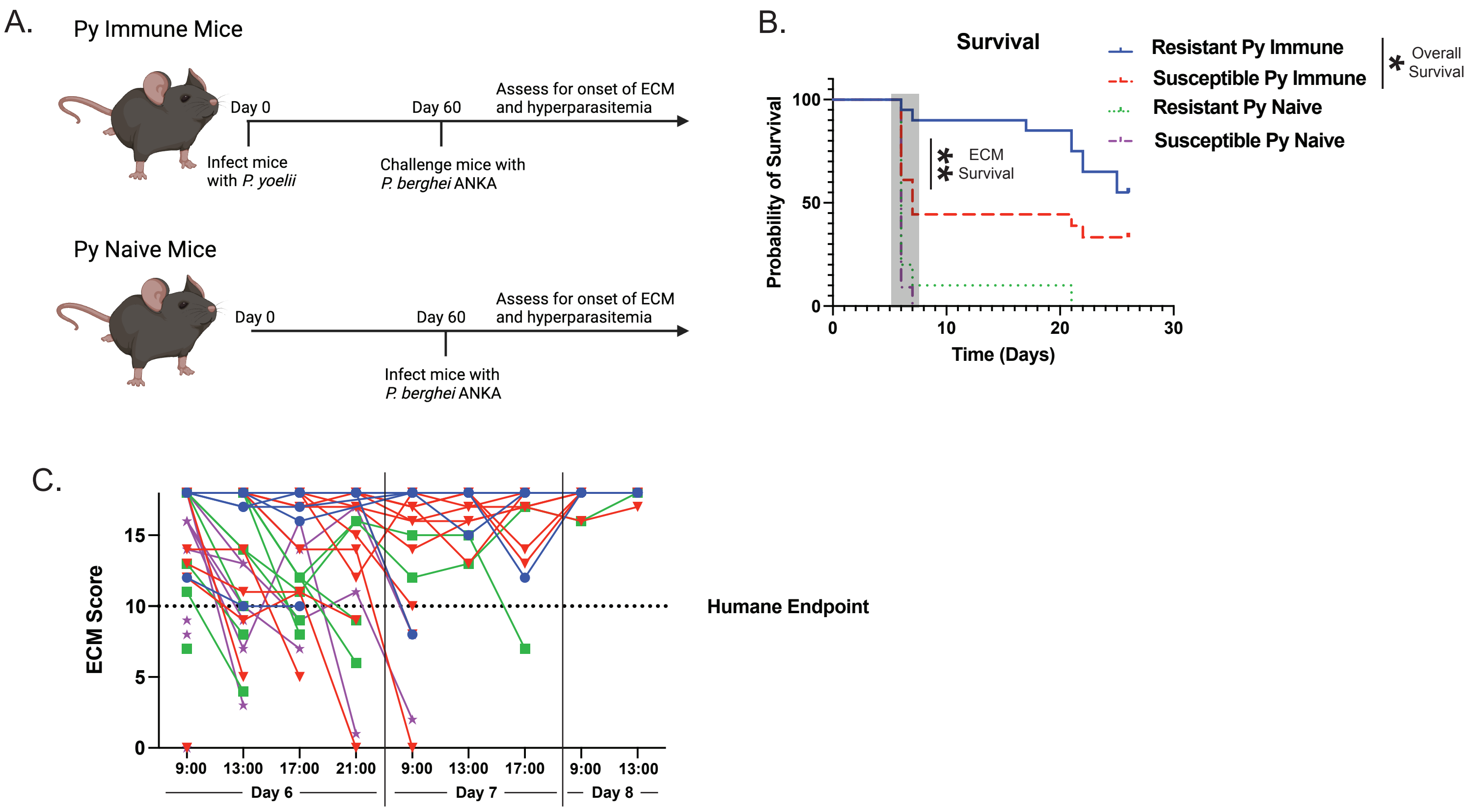

Supplemental Figure 3: Resistant mice are protected against *P. berghei* ANKA induced experimental cerebral malaria
(A) Schematic outlining experimental design. Mice that were previously infected with *P. yoelii* are *P. yoelii* (Py) immune. Mice that were not previously infected with *P. yoelii* are Py naïve. On day 60 post infection, all mice were infected with *P. berghei* ANKA infected red blood cells. Mice were assessed for the onset of experimental cerebral malaria (ECM) and hyperparasitemia.
(B) Survival curve following *P. berghei* ANKA infection. Deaths associated with *P. berghei* ANKA induced ECM occurred between days 6 and 8 post infection and are shaded in gray. Data (mean +/- SEM) are pooled from two independent experiments (n=20 Py immune resistant and susceptible mice n=10 Py naïve resistant and susceptible mice) and analyzed by Log-rank Mantel-Cox test.
(C) Graphical depiction of ECM scores during the ECM window. Time of routine monitoring is displayed based on the 24 hour clock. When a mouse reached a score of 10 or less they were humanely euthanized. Mice that were found dead or in unresponsive/comatose were given a score of 0. Recorded deaths include mice that had reached humane endpoint and mice that were found dead. With permission from the IACUC, several mice were kept alive for additional observation and experimentation after receiving a score of less than 10, but these mice were all humanely euthanized at later timepoints.
*p<0.05, **p<0.01, ***p<0.001, ****p<0.0001

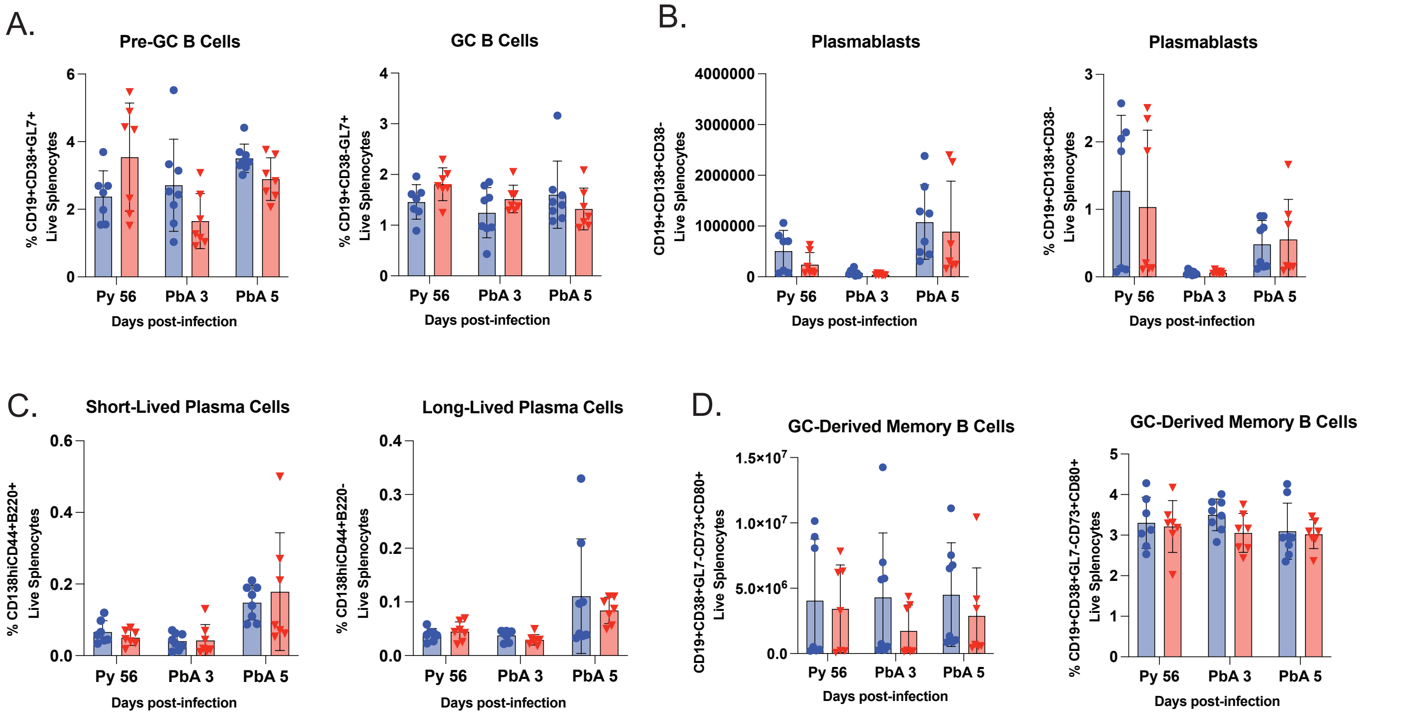

Supplemental Figure 4: Resistant and susceptible mice have a similar frequency of GC-associated B cells following *P. berghei* ANKA secondary challenge

(A and C) Percentage of Pre-GC B cells, GC B cells, SLPCs, and LLPCs present in the spleen at indicated time points post infection (n=7 or 8) (means +/- SEM) analyzed using Mann-Whitney test. Data are representative of two cumulative experiments.

(B and D) Number and percentage of GC-Derived MBCs (CD19+CD38+GL7-CD73+CD80+) and plasmablasts (CD19+CD138+CD38-) present in the spleen at indicated time points post infection (n=7 or 8) (means +/- SEM) analyzed using Mann-Whitney test. Data are pooled from two independent experiments.

*p<0.05, **p<0.01, ***p<0.001, ****p<0.0001

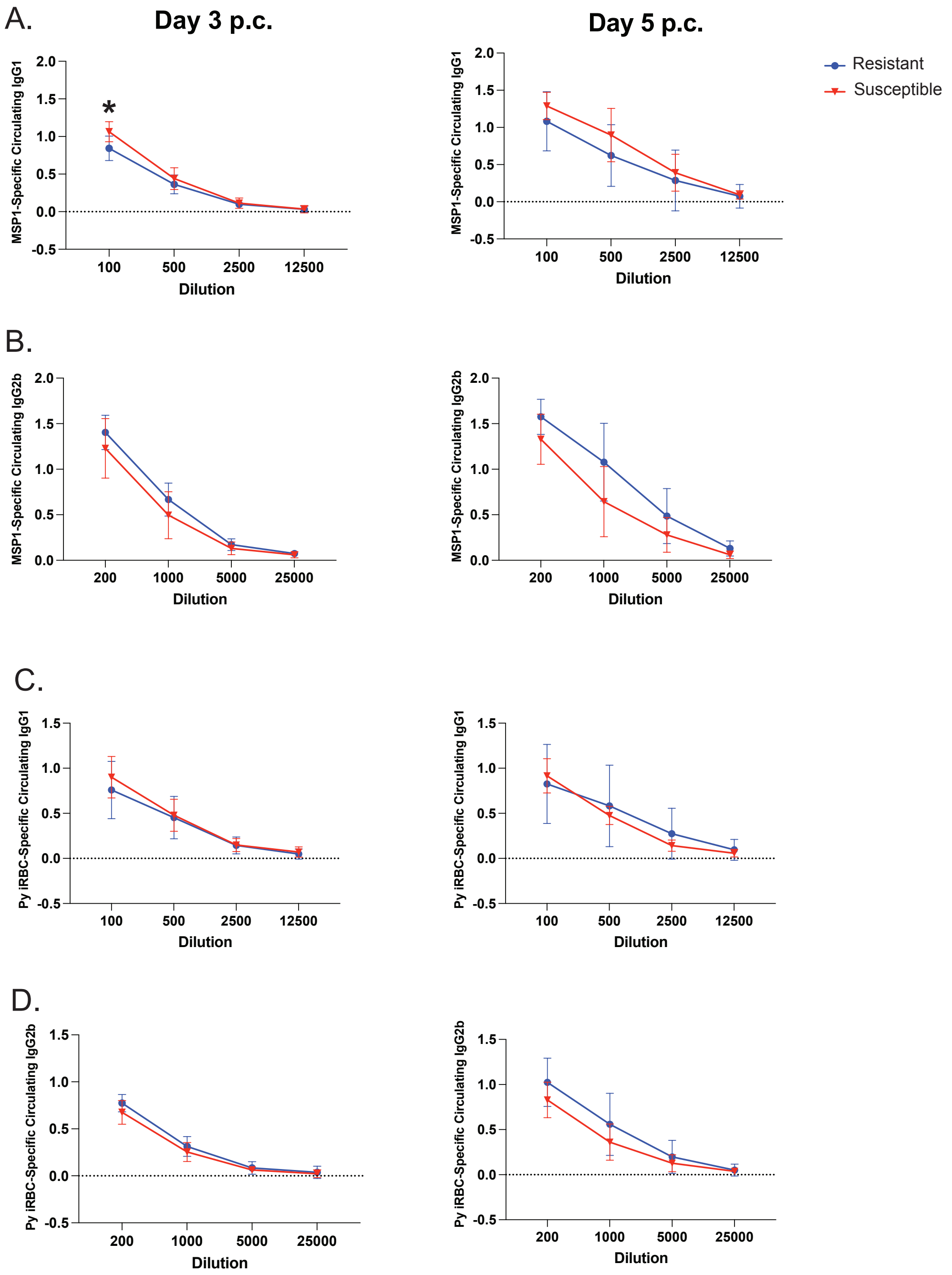

Supplemental Figure 5: Resistant and susceptible mice have similar circulating *P. yoelii*-specific antibody levels following *P. berghei* ANKA secondary challenge

(A and B) Sera was collected at days 3 and 5 post *P. berghei* ANKA challenge, diluted, and reacted against $MSP1_{19}$ coated plates to detect IgG1 and IgG2b antibodies by ELISA (n=7 or 8). Data (means +/- SEM) are pooled from two independent experiments and analyzed using Mann-Whitney test.

(C and D) Sera was collected at days 3 and 5 post *P. berghei* ANKA challenge, diluted, and reacted against lysed *P. yoelii* infected red blood cell protein-coated plates to detect IgG1 and IgG2b antibodies by ELISA (n=7 or 8). Data (means +/- SEM) are pooled from two independent experiments and analyzed using Mann-Whitney test.

*p<0.05, **p<0.01, ***p<0.001, ****p<0.0001

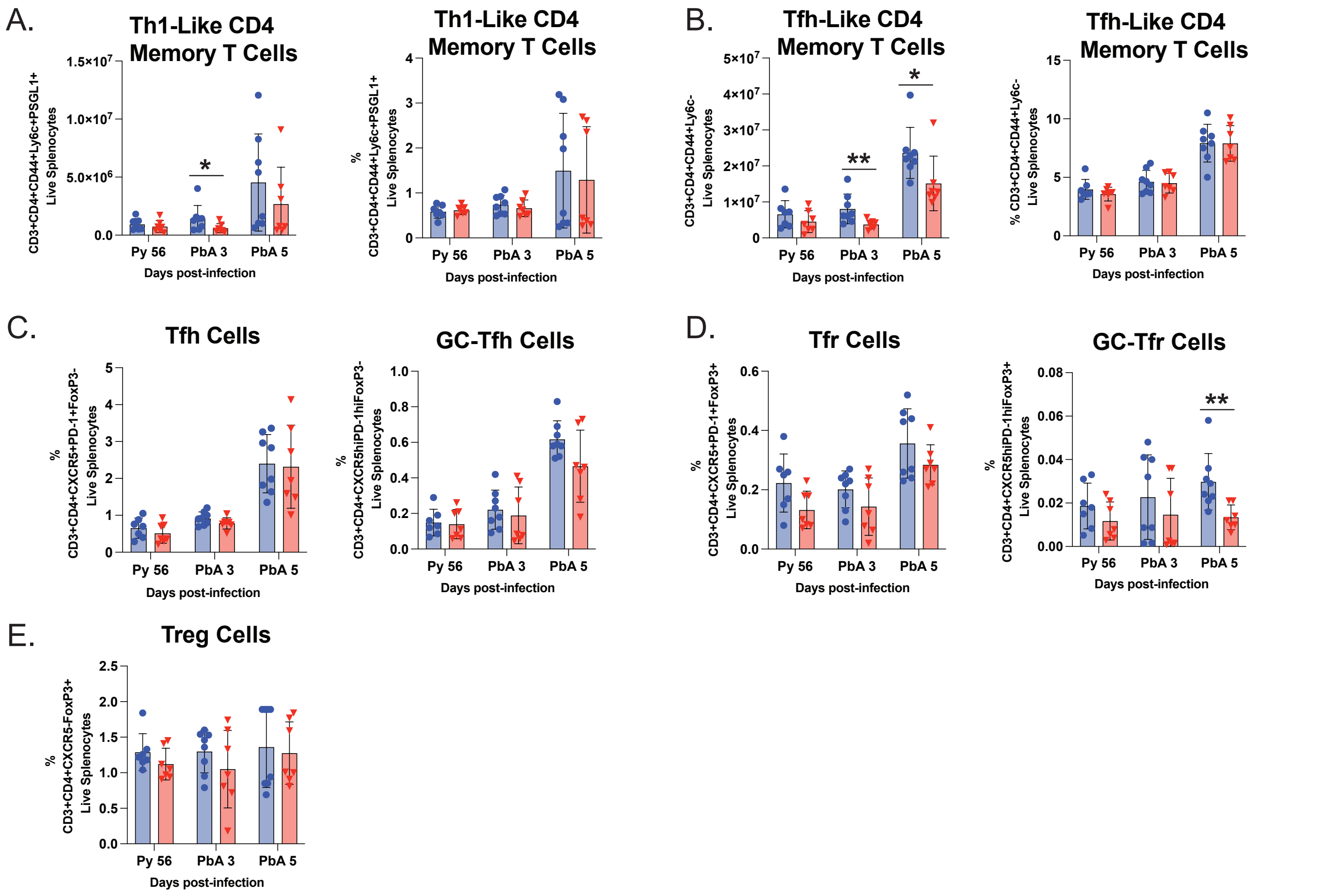

Supplemental Figure 6: Resistant and susceptible mice have a similar frequency of GC-associated T cells following *P. berghei* ANKA secondary challenge

(A and B) Number and percentage of Th1 like CD4 memory T cells (CD3+CD4+CD44+Ly6c+PSGL1+) and Tfh like CD4 memory T cells (CD3+CD4+CD44+Ly6c) present in the spleen at indicated time points post infection (n=7 or 8) (means +/- SEM) analyzed using Mann-Whitney test. Data are pooled from two independent experiments.

(C, D, and E) Percentage of Tfh cells, GC-Tfh cells, Tfr cells, GC-Tfr cells, and Tregs present in the spleen at indicated time points post infection with (n=7 or 8) (means +/- SEM) analyzed using Mann-Whitney test. Data are pooled from two independent experiments.

*p<0.05, **p<0.01, ***p<0.001, ****p<0.0001
